# Arthroscopic Superior Capsular Reconstruction With Long‐Head Biceps Tendon Rerouting Using Knotless Anchors

**DOI:** 10.1002/atn2.70302

**Published:** 2026-09-28

**Authors:** Julian Devigne, Vasileios Mitrousias, Gregory Cunningham

**Affiliations:** ^1^ CEPCO ‐ Shoulder and Elbow Center, Hirslanden, Clinique La Colline Geneva Switzerland; ^2^ Faculty of Medicine University of Geneva Geneva Switzerland

## Abstract

Irreparable rotator cuff tears remain a challenging condition to manage surgically, particularly in young and active patients without significant glenohumeral osteoarthritis. Several solutions have been proposed, and more recently, superior capsular reconstruction using the long head of the biceps tendon has shown promising results using an easily accessible autograft. In the present article, we describe an arthroscopic technique in which the long head of the biceps tendon is rerouted onto the greater tuberosity using knotless anchors for fixation of the tendon to greater tuberosity. This technique allows the stable long head of the biceps tendon compression to the bone and easy incorporation of the posterior cuff, resulting in a simple surgical construct. The advantage of this technique is the use of knotless anchors, which allows for fast, versatile, and bony‐preserving fixation technique.

## Graphical abstract

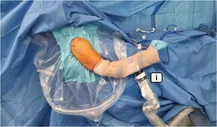

**VIDEO 1:** Arthroscopic superior capsular reconstruction using long‐head biceps tendon rerouting with knotless anchors in the left shoulder. The patient is positioned in the beach‐chair position with the left arm in slight abduction and neutral rotation. Arthroscopic visualization is obtained through a posterolateral viewing portal using a 30° arthroscope. After subacromial bursectomy and footprint preparation of the greater tuberosity, the long head of the biceps tendon is released distally and rerouted anterolaterally onto the greater tuberosity. A medial knotless anchor is inserted near the humeral head cartilage margin to secure the long head of the biceps tendon to bone. Remaining suture tapes are used to incorporate the posterior rotator cuff over the long head of the biceps scaffold. A second lateral knotless anchor is then inserted to complete fixation and enhance tendon compression and rerouting over the superior aspect of the greater tuberosity. Final construct stability is shown arthroscopically. Video content can be viewed at https://doi.org/10.1002/atn2.70302.

Massive, irreparable rotator cuff tears continue to represent a complex clinical problem, particularly in younger patients with minimal glenohumeral osteoarthritis. To prevent superior migration of the humeral head and restore dynamic centering, Mihata et al. were the first to describe a technique for superior capsular reconstruction (SCR) using the fascia lata.^1^ To avoid the use of allografts or autografts from other donor sites, Kim et al. described in 2018 a technique using the long head of the biceps (LHB) tendon rerouted to the greater tuberosity.^2^ This initial technique required lasso‐loop sutures and knot tying for fixation. Modifications of this technique have since emerged, such as the 1 described by Adrian et al., which further simplified the procedure by preserving the native attachment of the LHB, a variation commonly referred to as the bio‐SCR.^3^ The effectiveness of such techniques has been shown in several studies, and the efficacy and safety of the procedure are now proven.^4^, ^5^, ^6^

The aim of the present technique is to further simplify the procedure by using a low‐profile, 2‐anchor construct to maximize the bone–tendon interface and achieve strong compression and solid fixation of the LHB to the bone using the knotless tensioning anchor mechanism.

## SURGICAL TECHNIQUE

### Patient Selection

A massive and irreparable rotator cuff tear, an intact LHB tendon, and a satisfactory bone quality are the essential prerequisites for this technique (Video 1).

### Patient Positioning and Arthroscopic Setup

The procedure is performed under locoregional anesthesia with an interscalene block combined with sedation. The patient is positioned in the standard beach‐chair position with the arm in neutral rotation and slight abduction in a Trimano arm holder (Arthrex, Naples, FL) or an equivalent device (Figure 1). The procedure begins through a standard posterior portal with the introduction of a 3.5‐mm, 30° arthroscope.

**FIGURE 1:**
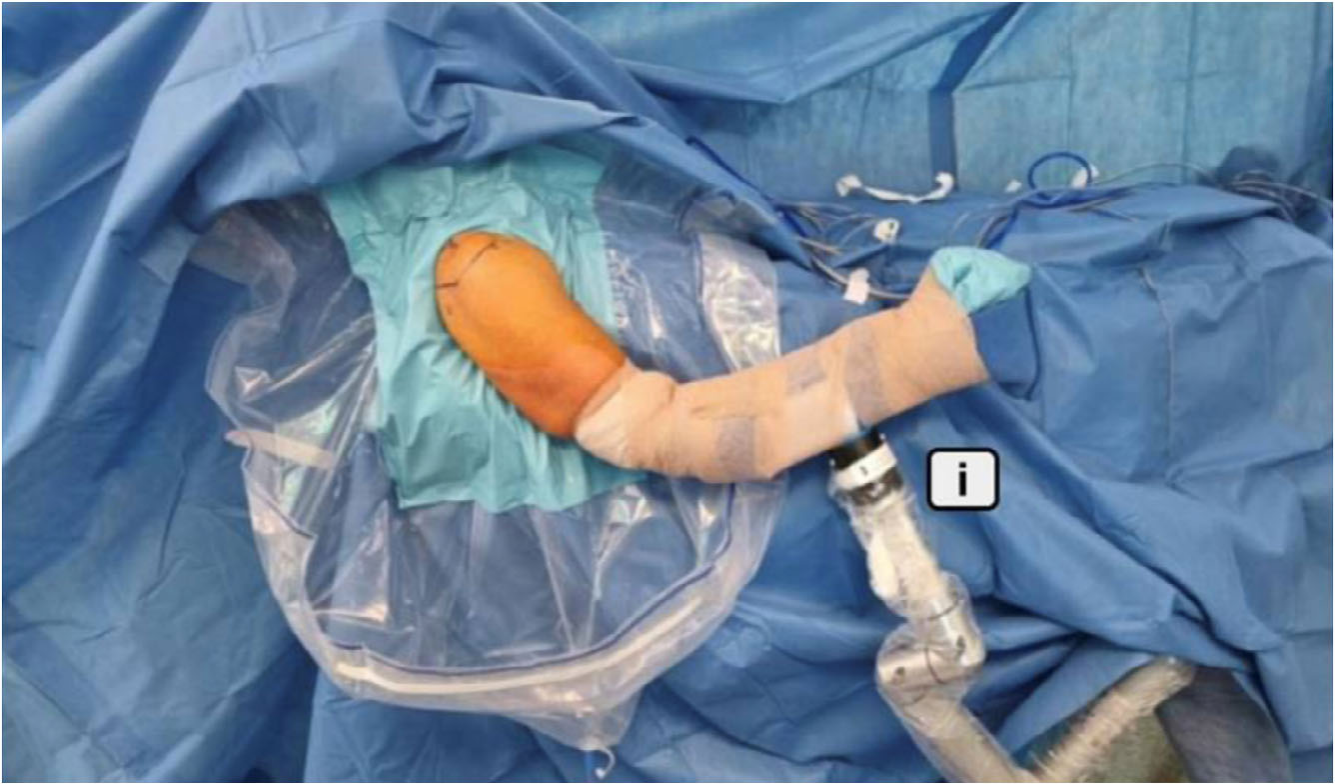
Operative room setup. The patient is placed in the beach‐chair position (45°‐60° of inclination) with the right arm in neutral rotation, slight abduction, and forward flexion assisted by a mechanical arm holder (i) (Trimano, Arthrex, Naples, Florida).

### Arthroscopic Evaluation

The LHB tendon is evaluated along with the subscapularis, supraspinatus, and infraspinatus tendons. Any repair on the subscapularis tendon is done at this stage. After switching to the subacromial space through the posterior portal, an anterolateral portal is created. The subacromial bursa is debrided, and the remaining posterior cuff tissue is mobilized using any of the various interval slide techniques to facilitate superolateral advancement. The maximal advancement location will determine where the LHB tendon will be transposed to the greater tuberosity.

### Footprint Preparation and Tendon Mobilization

The humeral footprint is freshened up with a 5.5 mm × 13 cm burr (Arthrex, Naples, FL) without decorticating the greater tuberosity to preserve a strong bony bed structure. The LHB tendon is released distally to allow mobilization and anterolateral rerouting toward the greater tuberosity.

### Fixation Technique

A posterolateral visualization portal is created. A first medial double‐loaded 2.6‐mm Fibertak knotless anchor (Arthrex, Naples, FL) is inserted through a dedicated portal at the predetermined location where the LHB tendon will be transferred, close to the humeral head cartilage margin (Figure 2). The shuttle suture is retrieved through the anterolateral canula, loaded on a Scorpion suture passer (Arthrex, Naples, FL), and passed through the LHB tendon (Figure 3). Then, the single suture strand is passed through the loop and shuttled through the biceps and into the anchor. It is tensioned to compress the biceps to the bone, locked, and cut flush. Two suture tapes with joined tails are now remaining. The joined tails are cut and 2 free suture tapes are available to use. The first tape is passed again through the LHB tendon, and the second is passed through the posterior rotator cuff (Figure 4). The 2 structures are then tied together, with the posterior rotator cuff overlaying the LHB scaffold. A second similar anchor is inserted laterally on the greater tuberosity following the exact same steps (Figure 5). This allows further redirection of the LHB tendon to the superior aspect of the greater tuberosity and provides an additional fixation point between the tendon and the posterior rotator cuff. The LHB can then be tenototomized distally or preserved, as in this case. The final construct is presented in Figure 6.

**FIGURE 2:**
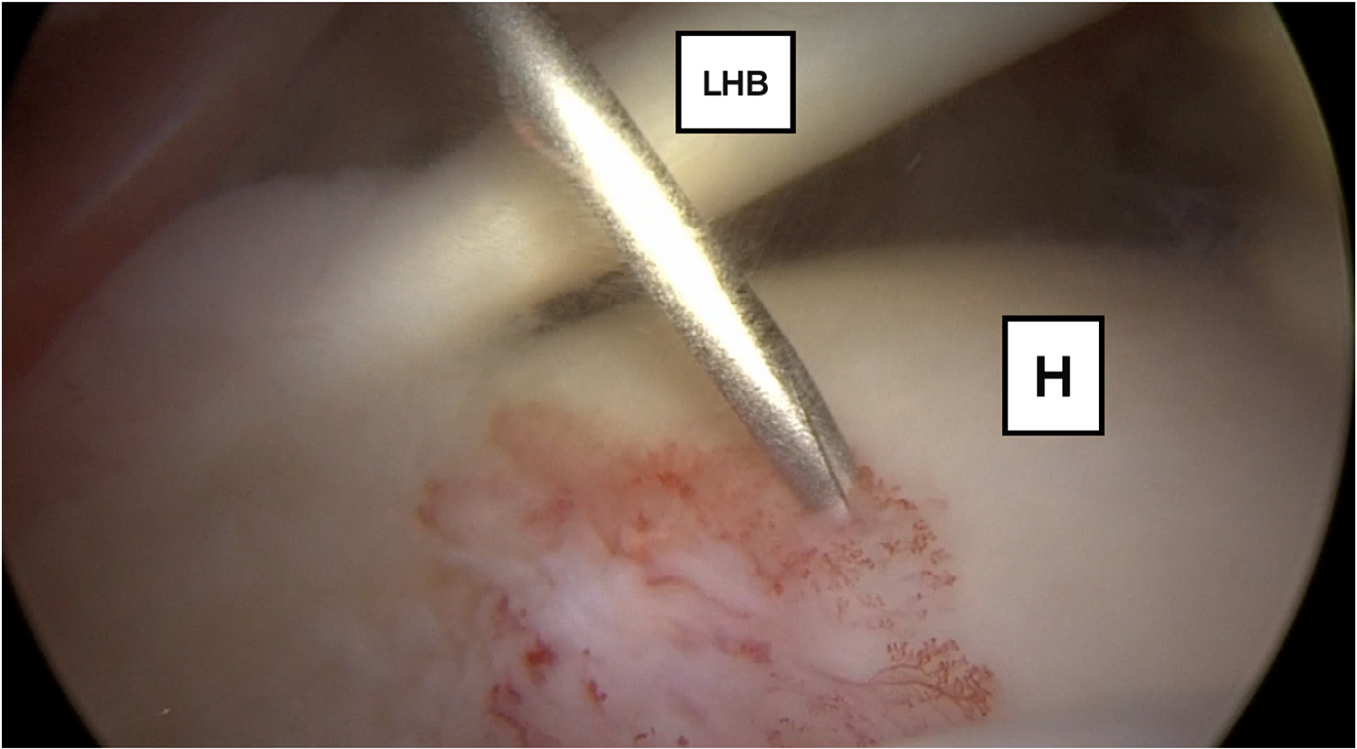
Arthroscopic view of a right shoulder in the beach‐chair position through the posterior portal, showing the LHB tendon and the placement of the first anchor at the junction between the articular surface and the humeral footprint. (H, humeral head; LHB, long head of the biceps.)

**FIGURE 3:**
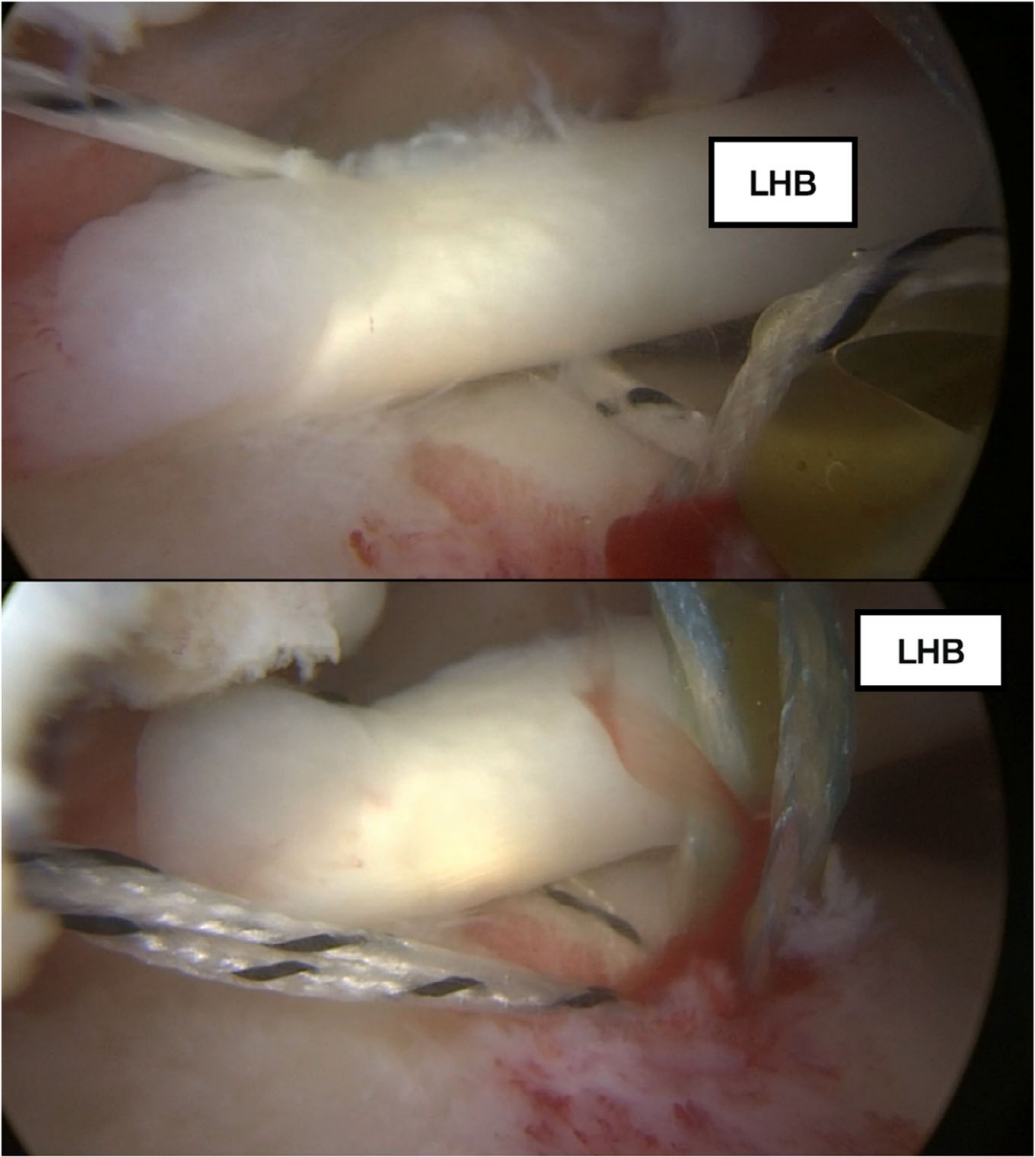
Arthroscopic view of a right shoulder in the beach‐chair position through the posterior portal, showing the passage of the knotless repair sutures through the biceps tendon. (LHB, long head of the biceps.)

**FIGURE 4:**
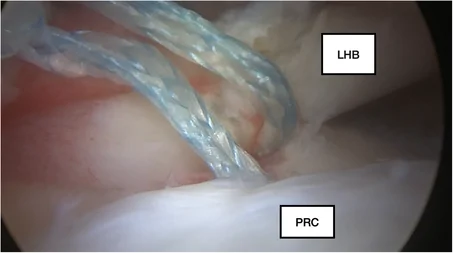
Arthroscopic view of a right shoulder in the beach‐chair position through the posterior portal, showing the LHB tendon and the posterior cuff being advanced superiorly and tied together with the 2 suture tapes. (LHB, long head of the biceps; PRC, posterior rotator cuff.)

**FIGURE 5:**
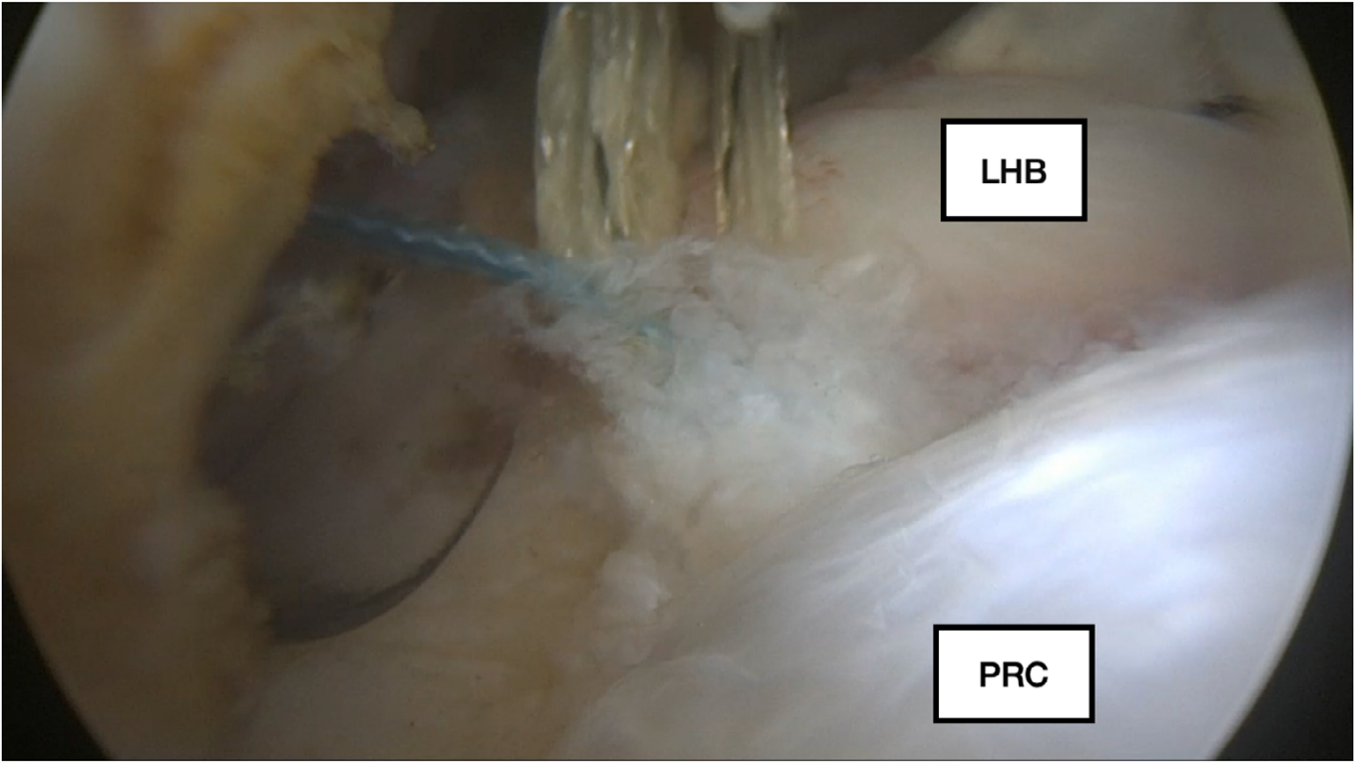
Arthroscopic view of a right shoulder in the beach‐chair position through the posterior portal, showing the placement of the second anchor more laterally to allow the anterior‐lateral rerouting of the LHB tendon. (LHB, long head of the biceps; PRC, posterior rotator cuff.)

**FIGURE 6:**
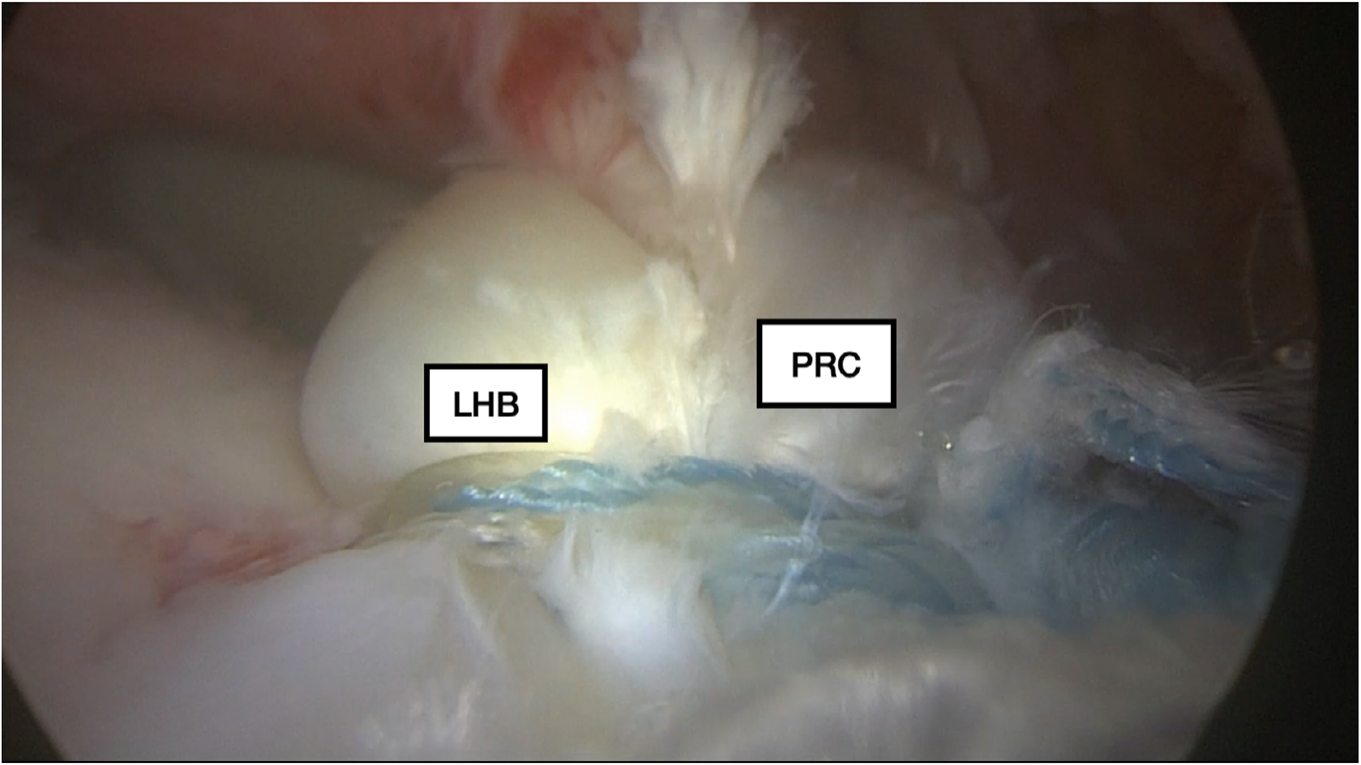
Arthroscopic view of a right shoulder in the beach‐chair position through the lateral portal, showing the final reconstruction from the lateral view, with anterior‐lateral deviation of the LHB tendon and its incorporation into the posterior rotator cuff. (LHB, long head of the biceps; PRC, posterior rotator cuff.)

### Postoperative Care

Postoperatively, the arm is immobilized in a neutral rotation sling for 4 to 6 weeks to reduce load on the posterior rotator cuff. Gentle self‐assisted and pendulum exercises are permitted. Physiotherapy begins with passive range of motion at 5 weeks. Pearls and pitfalls of the described technique are presented in Table 1.

**TABLE 1:** Pearls and Pitfalls

**Pearls**	**Pitfalls**
The use of a cannula facilitates the passage of instruments	A frayed or torn biceps can be an intraoperative finding which may prevent from carrying out this procedure and should be carefully examined on preoperative imaging
Mobilizing the biceps and posterior cuff to anchor insertion site helps evaluate tension of the construct	Anchor pullout may occur in softer bone on the greater tuberosity
Making an incision over the footprint allows perpendicular access for anchor insertion, thereby providing an optimal angle for anchor placement	

## DISCUSSION

The advantage of the presented technique is the use of knotless, low‐profile self‐punching anchors, avoiding the need for drilling and minimizing knot tying (Table 2). Studies, comparing knotted to knotless anchors in other shoulder procedures, have reported similar clinical outcomes and reduced operative time in favor of the knotless anchors.^7^, ^8^, ^9^ Biomechanically, conflicting results have been published because of the broad range of materials tested.^9^ However, in a recent study of Denard et al., comparing the fixation properties of knotted versus knotless constructs, knotless fixation using fibertape showed improved biomechanical performance and less variability.^10^ In a similar way, the presented knotless technique allows for a fast, strong, and secure fixation of the biceps while offering the versatility of incorporating the posterior rotator cuff using the additional suture tapes.

**TABLE 2:** Advantages and Disadvantages of the Presented Technique

**Advantages**	**Disadvantages**
This is a simple technique that allows compression of bone to tendon with locking mechanism inside the anchor avoiding bone‐suture slippage	Adequate bone quality is required as an anchor is directly implanted in the greater tuberosity
Small all‐suture anchors preserve bone stock	The presence of a healthy biceps tendon is necessary to perform the procedure
These anchors can be available off the shelf and less costly than other implants used for similar techniques such as dermal allografts	The attachment between the LHB tendon and the remaining posterior cuff still requires knot tying
	Required some experience with knotless anchors

LHB, long head of the biceps.

Another major advantage of this technique, compared with other options such as the subacromial balloon spacer or dermal allograft SCR, is its simplicity because it requires only 2 anchors that are readily available off the shelf^11^, ^12^ (Table 2). Furthermore, from a biological standpoint, simply rerouting the LHB tendon while keeping its proximal insertion allows it to act as a vascularized autograft and may potentially lead to better healing.

This technique has also certain limitations (Table 2). First, the use of knotless anchors implanted directly in the greater tuberosity requires adequate bone quality because the smaller anchor size may be less suitable in osteoporotic bone. Second, it requires the presence of a structurally preserved LHB tendon capable of being mobilized to the greater tuberosity to achieve the desired suspensory effect. However, the quality of the LHB tendon is a limitation for all available SCR techniques. Third, some prior experience with knotless anchors is required because their use can be challenging for inexperienced surgeons. Finally, the attachment between the LHB tendon and the remaining posterior cuff still requires knot tying, and a completely knotless construct has not been described yet.

Recent literature has further supported the role of SCR using the LHB tendon for the treatment of massive and irreparable rotator cuff tears. Gao et al. reported significant improvements in pain relief, shoulder mobility, and functional outcomes after SCR using the LHB tendon in patients with massive cuff tears.^13^ Similarly, Pastor et al. showed excellent clinical outcomes and low retear rates 1 year after cuff repair reinforced with LHB‐based SCR.^14^ In addition, Cañete San Pastor et al. described an arthroscopic technique using the LHB tendon to biologically reinforce supraspinatus repair and improve tendon healing.^15^ More broadly, the recent review by Lädermann et al. highlighted the growing interest and expanding indications for SCR in irreparable posterosuperior cuff tears.^16^ These recent studies further support the use of the LHB tendon as a local, vascularized, and cost‐effective autograft option in SCR procedures.

In conclusion, the use of self‐punching, knotless anchors in SCR using the LHB tendon can simplify the surgical procedure and provide a stable, secure fixation of the LHB tendon while permitting an easy incorporation of the posterior cuff to the final construct. Maintenance of the LHB glenoid attachment adds native, vascularized tissue in the repair, potentially increasing the healing potential of the construct. The technique requires no special instrumentation and can be a safe and versatile option for any irreparable rotator cuff tear with an intact LHB.

## DISCLOSURES

The authors (J.D., V.M., G.C.) declare that they have no known competing financial interests or personal relationships that could have appeared to influence the work reported in this article.

## References

[1] Mihata T , Lee TQ , Watanabe C , et al. Clinical results of arthroscopic superior capsule reconstruction for irreparable rotator cuff tears. Arthroscopy. 2013;29:459‐470. 10.1016/j.arthro.2012.10.022 23369443

[2] Kim YS , Lee HJ , Park I , Sung GY , Kim DJ , Kim JH . Arthroscopic in situ superior capsular reconstruction using the long head of the biceps tendon. Arthrosc Tech. 2018;7:e97‐e103. 10.1016/j.eats.2017.08.058 PMC5850885 29552475

[3] Adrian SC , Field LD . Biceps transposition for biological superior capsular reconstruction. Arthrosc Tech. 2020;9:e841‐e846. 10.1016/j.eats.2020.02.020 PMC7301374 32577361

[4] Cheppalli NS , Purudappa PP , Metikala S , et al. Superior capsular reconstruction using the biceps tendon in the treatment of irreparable massive rotator cuff tears improves patient‐reported outcome scores: A systematic review. Arthrosc Sports Med Rehabil. 2022;4:e1235‐e1243. 10.1016/j.asmr.2022.04.003 PMC9210484 35747666

[5] Kitridis D , Yiannakopoulos C , Sinopidis C , Givissis P , Galanis N . Superior capsular reconstruction of the shoulder using the long head of the biceps tendon: A systematic review of surgical techniques and clinical outcomes. Medicina. 2021;57:229. 10.3390/medicina57030229 PMC8000769 33801508

[6] Shin KH , Jang IT , Han SB . Outcomes of superior capsular reconstruction using the long head of the biceps tendon in large to massive rotator cuff tears: A meta‐analysis and systematic review. J Clin Med. 2024;13:1052. 10.3390/jcm13041052 PMC10889218 38398365

[7] Arner JW , De Groot J , Bradley JP . Arthroscopic SLAP IIb repair using knot‐tying versus knotless suture anchors: Is there a difference? Arthroscopy. 2018;34:e26. 10.12788/ajo.2018.0101 30650165

[8] Zumstein MA , Mueller‐Lebschi J , Rojas T . *Editorial commentary*: No difference in knotted versus knotless anchors for labral repair in the shoulder. Arthroscopy. 2021;37:1322. 10.1016/j.arthro.2021.01.052 33812530

[9] Matache BA , Hurley ET , Kanakamedala AC , et al. Knotted versus knotless anchors for labral repair in the shoulder: A systematic review. Arthroscopy. 2021;37:1314‐1321. 10.1016/j.arthro.2020.11.056 33307150

[10] Denard PJ , Adams CR , Fischer NC , Piepenbrink M , Wijdicks CA . Knotless fixation is stronger and less variable than knotted constructs in securing a suture loop. Orthop J Sports Med. 2018;6:2325967118774000. 10.1177/2325967118774000 PMC5964856 29845084

[11] Cuéllar A , Cuéllar R . *Editorial commentary*: Shoulder superior capsular reconstruction with dermal allograft is useful for active patients with massive irreparable rotator cuff tear. Arthroscopy. 2021;37:497‐498. 10.1016/j.arthro.2020.12.182 33546788

[12] Rondon AJ , Farronato DM , Pezzulo JD , Abboud JA . Irreparable massive rotator cuff tears: Subacromial balloon surgical technique. Arthrosc Tech. 2022;12:e421‐e432. 10.1016/j.eats.2022.08.048 PMC10066046 37013017

[13] Gao Q , Qiao Y , Guan Y , et al. Superior capsular reconstruction using the long head of the biceps to treat massive rotator cuff tears improves patients shoulder pain, mobility and function. Knee Surg Sports Traumatol Arthrosc. 2023;31:4559‐4565. 10.1007/s00167-023-07489-7 37338624

[14] Pastor PCS , Ramos MIP , Roig AG , Safont JA . Superior capsular reconstruction with the long head of the biceps tendon achieves excellent clinical results and low rotator cuff rerupture rates one year after cuff repair surgery. Int Orthop. 2024;48:2121‐2128. 10.1007/s00264-024-06223-3 38796811

[15] Cañete San Pastor P , Prosper Ramos I , Garcia Roig A . Arthroscopic superior capsular reconstruction with the long head of the biceps tendon to reinforce the repair of a supraspinatus tear and increase healing. Arthrosc Tech. 2023;12:e1457‐e1465. 10.1016/j.eats.2023.04.017 PMC10466433 37654873

[16] Lädermann A . Superior capsular reconstruction for irreparable posterosuperior rotator cuff tears. Orthop Traumatol Surg Res. 2024;110:103758. 10.1016/j.otsr.2023.103758 37972708

